# Photoreceptor structure and function is maintained in organotypic cultures of mouse retinas

**Published:** 2010-06-26

**Authors:** Mausumi Bandyopadhyay, Bärbel Rohrer

**Affiliations:** 1 Department of Neurosciences, Division of Research, Medical University of South Carolina, Charleston, SC; 2 Department of Ophthalmology, Medical University of South Carolina, Charleston, SC

## Abstract

**Purpose:**

Retina organ cultures can be used as a valuable tool to study retina development ex vivo. Comparison between culture methods has revealed that timing the start of the culture and the presence of the retinal pigment epithelium (RPE) are critical for the development of the rods and cones, which are the two types of photoreceptors; rods can develop in the absence of the RPE, cones cannot. One of the necessary compounds produced by the RPE and essential for cone development and survival is the chromophore 11-*cis* retinal. Here, we further examined rod and cone development, chromophore production by the RPE, and photoreceptor signaling to the inner retina under organ culture conditions.

**Methods:**

Retina-RPE cultures were prepared from 7-day-old C57BL/6 pups and maintained in culture for 11 days. Rod and cone structure was analyzed by immunohistochemistry, and cell-specific mRNA expression was analyzed by quantitative real-time PCR. We quantified 11-*cis* retinal spectrophotometrically by measuring rhodopsin. Signal transmission in the rod pathway was studied by analyzing *c-fos* expression in the inner retina in response to stroboscopic illumination.

**Results:**

In retina-RPE cultures analyzed after 11 days in culture, rod and cone numbers exhibited a similar ratio to those observed in the intact animal. Although photoreceptor outer segments were shorter when grown ex vivo, membrane proteins, such as cone opsin and transducin, were localized appropriately to the outer segment. Relative 11-*cis* retinal production ex vivo plateaued after 7 days in culture, resulting in approximately 30% of the in vivo level by day 11. The retinas responded to prolonged stroboscopic illumination with the normal nuclear expression of c-fos in cells in the inner retina.

**Conclusions:**

Mouse retinal structure is maintained in retina–RPE organ cultures. The RPE in organ cultures produces sufficient amounts of 11-*cis* retinal to promote cone development and support signal transmission in the rod pathway. Organ cultures may be a powerful low-throughput screening tool to identify novel agents to promote photoreceptor cell survival and signaling.

## Introduction

Mouse retinas are amenable to growth in culture using either the isolated retina [1] or retina-retinal pigment epithelium (RPE) co-cultures [2]. Under both culture conditions, rod photoreceptor development is supported, although outer segment development and maintenance is impaired in the absence of the RPE [1]. No peanut agglutinin (PNA) lectin-positive cone sheets or cone opsin-positive cone outer segments could be detected in a retina-only culture (Rohrer, unpublished results, 2007), indicating that cone development requires the presence of the RPE. This is in contrast to the retina–RPE co-cultures, which do support cone development and outer segment maintenance. However, timing of the cultures seems to be critical; starting organ cultures at postnatal day 2 (P2) results in the development of short-wave (ultraviolet [UV])-sensitive, but not middle-wavelength (MWL)-sensitive cones [3]; the development of MWL cones is only supported in vitro when explants are harvested from pups P3 or older [4]. Thus, explants starting from P3 or older should be used to generate a mouse retina with all three types of photoreceptors.

For organ cultures to be a useful tool to study or manipulate photoreceptor development and function, baseline parameters first have to be established. Here, we have asked four related questions addressing different aspects of the photoreceptor cells: cell numbers, subcellular localization of cell-type specific proteins, pigment content, and postsynaptic signaling. Pertaining to the first aspect, rods outnumber cones by a ratio of 30:1 in the mouse in vivo [5], and a similar ratio is predicted under organ culture conditions. Second, regarding subcellular localization of cell-type specific proteins, early in development the rod and cone apoproteins are distributed throughout the entire cell, whereas cell membrane labeling disappears around the onset of vision when the cell matures [6-9]. This restriction of cone opsin to the outer segment is dependent upon the presence of 11-*cis* retinal [10-15], whereas the restriction of rod opsin is 11-*cis* retinal independent [16]. As a consequence, cone cell death is associated with a lack of trafficking of cone outer segment proteins to the outer segment [10]. Here, we have asked whether the RPE in RPE–retina co-cultures generates sufficient 11-*cis* retinal to promote cone maturation. However, in this context it is important to consider that in isolated RPE cultures, the expression of RPE65, the isomerohydrolase required for the production of the chromophore 11-*cis* retinal [17], appears to be sensitive to culture conditions [18,19], age [20], and source of tissue [21]. Third, in the same context, levels of RPE65 might be the rate-limiting step for the formation of pigment as well as opsin expression. Redmond and colleagues have shown that mice with one copy of *RPE65* have significantly reduced levels of rhodopsin and opsin [16]. Fourth, as a final test for functionality, we tested whether signal transmission occurs in the rod pathway under organ culture conditions. To this end, we took advantage of the observation that stroboscopic illumination induces transcription factor expression (c-fos) in the inner retina in several different species [22-24], a mechanism that requires a functional rod–ribbon synapse [23].

Taken together, we have tested and shown that commonly used organ culture conditions generate a tissue environment that supports normal rod and cone development, 11-*cis* retinal production sufficient for cone development and maintenance, as well as signaling from the photoreceptors to the inner retina.

## Methods

### Animals

C57BL/6 mice were generated from breeding pairs obtained from Harlan Laboratories (Indianapolis, IN). Animals were housed in the Medical University of South Carolina (MUSC) Animal Care Facility under a 12 h:12 h, light–dark cycle, with access to food and water ad libitum. All experiments were performed in accordance with the Association for Research in Vision and Ophthalmology (ARVO) Statement for the Use of Animals in Ophthalmic and Vision Research and were approved by the MUSC Animal Care and Use Committee.

### Retinotypic cultures

All chemicals and reagents used for organ cultures were tissue culture grade and were purchased from Invitrogen (Carlsbad, CA). Retina–RPE cultures were grown by means of the interface technique according to published protocols [1,2,25] with modifications. All preparations were performed under a laminar flow hood. In short, pups were deeply anesthetized by hypothermia and were then decapitated. A total of 50 pups was used for the retinotypic cultures, and 25 as in vivo controls. Heads were rinsed in 70% ethanol, and eyeballs were collected and placed in ice-cold Hanks balanced salt solution plus glucose (6.5 g/l). To collect the retina with RPE, eyes were incubated in 1 ml of media containing cystein (0.035 mg) and papain (20 U) at 37 °C for 15 min. Enzymatic activity was stopped by adding media plus 10% fetal calf serum. The anterior chamber was removed, followed by the lens and vitreous. Using a pair of #5 forceps, the retina with the RPE attached was then carefully dissected free from the choroid and sclera. Relaxing cuts were made into the retina–RPE sandwich to flatten the tissues. The tissues were then transferred to the upper compartment of a Costar Transwell chamber (Fisher Scientific; Waltham, MA) using a drop of Neurobasal medium, with the RPE layer placed face down. The drop of fluid was used to flatten the retina by gently spreading the drop of liquid with the fused end of a glass Pasteur pipette. Neurobasal media supplemented with 1% N1 and 2% B-27 supplements (Invitrogen) were placed in the lower compartment. The cultures were kept in an incubator (5% CO_2_, balanced air, 100% humidity, at 37 °C), and the medium was changed every 2 days under dim red light. No antimicotics or antibiotics were required.

### Immunohistochemistry

Retina cultures were fixed in 4% paraformaldehyde within 10 min of removal from the dark incubator. For sections, tissues were cryoprotected in 30% sucrose, frozen in TissueTek optimum cutting temperature formulation (OCT; Fisher Scientific, Waltham, MA), and cut into 14-μm cryostat sections [23,25,26]. After the slides were washed in phosphate buffer solution (PBS; 2.7 mM KCl, 138 mM NaCl, 6.6 mM Na_2_HPO_4_(H_2_O), 1.8 mM KH_2_PO_4_), they were blocked with 10% normal goat serum (Jackson ImmunoResearch Laboratories, West Grove, PA) and 3% BSA (in PBS containing 0.4% Triton X). Tissues were incubated overnight in blocking solution containing the antibodies of interest, followed by incubation with the appropriate fluorescent-labeled secondary antibody for 2 h (Molecular Probes, Carlsbad, CA). Control experiments included omission of primary antibody and observation of singly-labeled slides through the appropriate filter set. Sections were mounted and analyzed by confocal microscopy (Leica, Bannockburn, IL) using identical settings for all slides. Images were imported into Adobe Photoshop software (Adobe Systems, San Jose, CA) for further analysis. The following primary antibodies were used in this study: rhodopsin (1D4; Santa Cruz Biotechnology, Santa Cruz, CA [27,28]), mouse ultraviolet (UV) and middle wavelength (MWL) opsin [10,29], mouse cone arrestin (mCAR); [30], Gnat2 (Santa Cruz Biotechnology [31]), and c-fos (Santa Cruz Biotechnology [23,32]). All these antibodies have been shown to specifically recognize the antigen they were raised against (see provided references); for convenience we will use “antigen” to mean “antigen-immunoreactivity.”

### Pigment measurements

Pigment measurements were performed on samples from age-matched animals and retina explant cultures. Mice were dark-adapted overnight before tissue collection, and explant cultures were manipulated under dim red light. All procedures were performed under dim red light to prevent photobleaching. Endogenous levels of pigment were determined by extraction of a homogenate of two retinas as described previously [33] with modifications. In short, tissues were homogenized in 500 μl PBS containing 1X protease inhibitor (Sigma-Aldrich; St. Louis, MO). Samples were centrifuged (27,000× g, 15 min), the supernatant discarded and the remaining pellet solubilized in 1% dodecylmaltoside (in sodium phosphate buffer, pH 7.4). The sample was shaken at 4 °C for 2 h, centrifuged (88,000× g for 10 min), and measured on a Softmax Pro spectrophotometer (Varian; Palo Alto, CA). The difference spectra were determined from measurements before and after bleaching with white light. The concentration of rhodopsin was calculated using the following extinction coefficient: ε (rhodopsin)=40,600 l·mol^−1^·cm^−1^ [34].

### Quantitative real-time polymerase chain reaction

Real-time PCR was performed as previously described [35]. In short, RNA (2 μg each) were used to generate cDNA in reverse-transcription reactions (Invitrogen). PCR amplifications were conducted using the QuantiTect Syber Green PCR Kit (Qiagen) with 0.01 U/µl AmpErase® UNG enzyme (Applied Biosystems; Foster City, CA) to prevent carryover contamination, using the following primers: 5′- GCT ACA GCT TCA CCA CCA CA-3′, reverse 5′- TCT CCA GGG AGG AAG AGG AT-3′; UV opsin, forward 5′- TTG GGC TCT GTA GCA GGT CT-3′, reverse 5′- CAA GTA GCC AGG ACC ACC AT-3′; MWL opsin, forward 5′- CTC TGC TAC CTC CAA GTG TGG-3′, reverse 5′- AAG TAT AGG GTC CCC AGC AGA-3′; cone transducin (*Gnat2*), forward 5′- GCA TCA GTG CTG AGG ACA AA-3′, reverse 5′- CTA GGC ACT CTT CGG GTG AG-3′; and *Rpe65*, forward 5′- TGC ATG CAC AGA GAC CAA CT-3′, reverse 5′- CAG TGG CAC CAT TGA CAG AA-3′. Quantitative values were obtained from the cycle number (C_t_ value) to establish the fold difference in gene expression between the treated and untreated retina cultures [33].

### Stroboscopic illumination

Stroboscopic illumination was used to study light-induced gene expression in the inner retina [22,23]. For exposure to stroboscopic light, we used a battery-powered 7500 V Xenon strobe light (Xenon Strobe ESL I; Landfall Navigation, Stamford, CT) that presented flashes of white light at approximately 1 Hz. The light source was placed 25 cm above the culture plates exposing tissues to the flashing light for 2 h to allow for induction of protein expression. As a positive control, animals that were dark-adapted overnight were exposed in the same manner. Cultures and eyes were collected and processed as described above.

### Statistics

Data are expressed as means± standard deviation (SSD). Statview software (SAS Institute; Cary, NC) was used for statistical analysis. Comparison of two conditions was performed using the Student *t* test, with p<0.05 accepted as significant.

## Results and discussion

### Retinal development is recapitulated in organ culture

**Figure 1:**
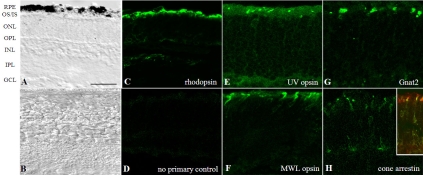
Establishment of a mouse retina–retinal pigment epithelium (RPE) explant system. **A**: The bright-field micrograph of a C57BL/6 mouse retinal explant placed in culture at postnatal day 7 (P7; which is called days in vitro 0, DIV0) and analyzed at DIV11 (P18=P7+11DIV) shows that normal retinal layers are formed and maintained in situ. **B**: A P18 C57BL/6 mouse retina is provided for comparison. In culture, rhodopsin (**C**), ultraviolet (UV) opsin (**E**), middle-wavelength (MWL) opsin (**F**), and cone transducin (**G**) are trafficked properly to the photoreceptor outer segment. **H**: Cone arrestin is present in the inner segments and cone pedicles, as described for light-adapted retinas [30]; a double labeling (inset of panel **H**) of cone arrestin with PNA-lectin is provided to allow for visualization of inner and outer segments. **D**: No-primary antibody control is provided for nonspecific staining of the secondary antibodies. Images are representative examples from three to five different litters. Abbreviations: GCL, ganglion cell layer; INL, inner nuclear layer; IPL, inner plexiform layer; IS, inner segments; ONL, outer nuclear layer; OPL, outer plexiform layer; OS, outer segments. Scale bar 50 μm.

We established a retina–RPE explant culture to analyze rod and cone development under controlled conditions. The defined media used here (Neurobasal media, N1 and B-27 supplements) contains vitamin A levels comparable to those found in mouse serum (Invitrogen, personal communication, 2007; and [34]), providing substrate for the generation of 11-*cis* retinal. Tissues were placed in organ cultures starting at P7, a time point when inner retinal layer formation is complete, but rod precursors actively migrate through the outer plexiform layer into the outer photoreceptor layer, a process that is complete between P10 and P12 [23]. After P10, further maturation of the retina includes the growth of the outer segments and refinement of synaptic connections. Analysis was performed after 11 days in vitro (DIV; P7+11DIV) and compared to P18 in vivo retinas. As shown by others (e.g., [1,2]), early postnatal retinas complete retinal development, in vitro resulting in an anatomic configuration within these explants that is comparable to that of retinal tissue in vivo (Figure 1A,B). However, photoreceptor outer segments are shortened and retinal ganglion cells are gradually lost when growing RPE–retina in organ culture; phenomena recognized by laboratories growing these kinds of cultures (e.g., [1,2]).

Rod development occurs independent of the presence of RPE [1], and rod opsin localization to the outer segments is independent of the presence of 11-*cis* retinal [16]. However, in an earlier study we described the requirement of 11-*cis* retinal for cone opsin expression and cone survival [35] and later showed that 11-*cis* retinal is necessary for the localization of cone opsin and cone outer segment membrane proteins, such as cone transducin (Gnat2), to the cone outer segments of the mouse retina [10-13]. Our ex vivo RPE–retinal explants, under defined experimental culture condition, mimic in vivo retinas. As reported previously [2-4], immunohistochemical analysis of retinal explants using antibodies against the 1D4 epitope of rhodopsin or mouse cone opsins (UV and MWL opsin) revealed that rhodopsin (Figure 1C), as well as the two cone opsins (Figure 1E,F), localize properly to the rod and cone outer segments in P7+11DIV cultures, rod and cone outer segments, respectively. In addition, we found normal localization of cone transducin in the cone outer segments (Gnat2; Figure 1G), and cone arrestin is present in the cone inner segments and pedicles, as described for light-adapted retinas [30] (Figure 1H). Double labeling of cones with cone arrestin and PNA lectin confirmed the presence of cone arrestin in the cone inner segment (see inset, Figure 1H).

**Figure 2:**
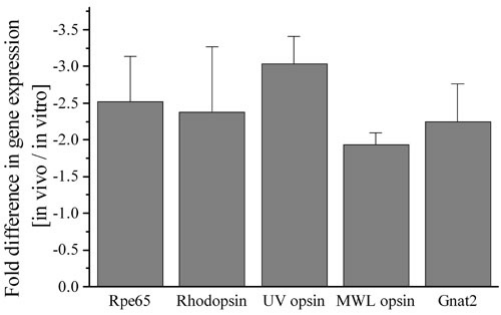
Quantification of retinal pigment epithelium-(RPE) and photoreceptor-specific gene expression. Quantitative real-time-polymerase chain reaction (QRT–PCR) was performed, comparing retina–RPE samples from intact mice and those grown in organotypic cultures starting from postnatal day 7. Analyzed were expression levels of Rpe65 (the rate-limiting enzyme required to generate 11-*cis* retinal), one rod-specific gene (rhodopsin), and three cone-specific genes (ultraviolet [UV] and middle-wavelength [MWL] opsin and cone transducin [*Gnat2*]). Explants contain 2-fold to 3-fold less mRNA for the tested genes at P7+11DIV than age-matched (P18) tissues that developed in vivo. Data are represented as mean±standard deviation of three different sets of experiments.

A normal in vivo mouse retina at P18 contains on average 10±0.5 vertical and 95.2±3.1 horizontal rows of rods and approximately 30.7±3.5 Gnat2-positive cone outer segments, per 420 μm window. In comparison, at P7+11DIV, the retina contains 6.7±0.2 vertical and 77.2±2.2 horizontal rows of rods (p<0.01) and approximately 14.2±2.4 Gnat2-positive cone outer segments (p<0.05). To establish the ratio of rods:cones per window in the central retina, the number of rods was obtained by multiplying the number of horizontal rows of photoreceptor nuclei by the height in nuclei of these rows. Using this comparison we confirmed that the ratio of rods:cones is close to 30:1 in retinas collected from both in vivo and in vitro conditions (in vivo, 971±37 rods and 30.7±3.5 cones, ratio 31:1; in vitro, 516±25 rods and 14.2±2.4 cones, ratio 36:1). Although the rod:cone ratio was approximately 15% higher in the in vitro condition, this difference was not statistically significant, largely due to the number of mice tested under each condition (n=6). The reduced number of rods and cones as well as the shortened outer segments present in cultured photoreceptors is reflected by 2-fold to 3-fold lower levels of rhodopsin, UV and MWL opsin, and *Gnat2* mRNA levels (see Figure 2).

Taken together, using a snapshot at P7+11DIV as reported by others, retinal layers are retained in organ culture [1,3]. While a close to normal rod:cone ratio is maintained in retina–RPE organ cultures from day 7, the overall number of photoreceptor cells is significantly reduced. And although rod and cone outer segments are shorter in vitro than under in vivo conditions, outer segment membrane proteins are nevertheless restricted to the outer segments after 11 days in culture.

### Light-mediated signaling in organ culture

**Figure 3:**
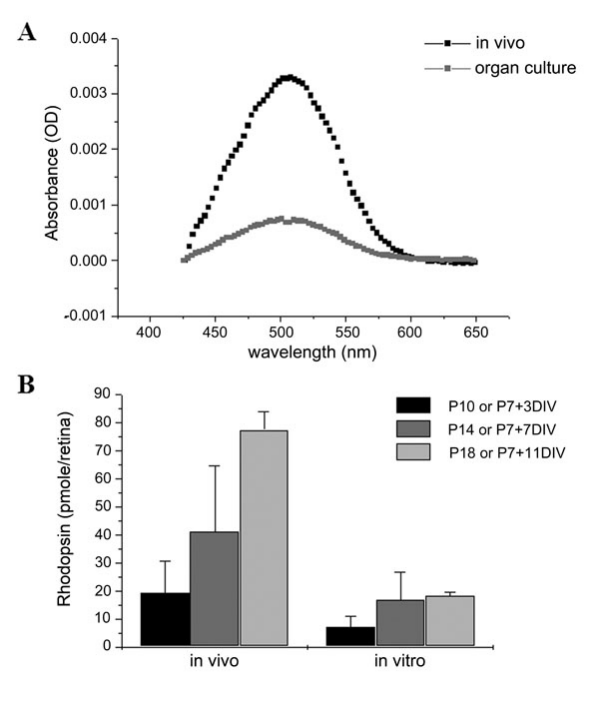
Quantification of 11-*cis* retinal. Since approximately 97% of all available 11-*cis* retinal in the mouse eye is bound to rhodopsin, chromophore was indirectly quantified measuring rhodopsin levels in the retina-retinal pigment epithelium (RPE) fraction. **A**: Example of difference spectra for P18 retinas from dark-adapted intact animals and retinas grown in organ culture starting at postnatal day 7 maintained in complete darkness. **B**: The amounts of rhodopsin were quantified from wild-type littermates and retinal explants at different postnatal days. Explants contain approximately 40% of the amount of rhodopsin of the in vivo animals at P7+7DIV, and approximately 25% at the later age-group analysis. Data are represented as mean±standard deviation of three to four different sets of experiments.

Normal cone outer segment protein trafficking implies that the photoreceptors have available a significant amount of 11-*cis* retinal. To determine the amount of 11-*cis* retinal generated in these cultures, the amount of rhodopsin was assayed in dark-adapted littermates and retinal explants at different postnatal days (Figure 3A,B). As more than 97% of 11-*cis* retinal is bound to the opsin apoprotein in the mouse retina, the concentration of rhodopsin is a good approximation of the concentration of 11-*cis* retinal generated in the RPE [36]. The rhodopsin concentration in explants gradually increased with time in culture, reaching a plateau of approximately 20 pmol/retina at P7+7DIV (Figure 3B). This is in comparison to the rapid increase in 11-*cis* retinal in the in vivo retina, which reaches levels of 77±6.6 pmol/retina at P18 (Figure 3B). Since RPE65 appears to be the rate-limiting enzyme required for the production of 11-*cis* retinal [37], *Rpe65* mRNA was quantified using Quantitative reverse transcriptase (QRT)–PCR. At P7+11DIV, *Rpe65* mRNA levels in organ cultures were approximately 2.5-fold lower than in age-matched in vivo littermates (Figure 2).

**Figure 4:**
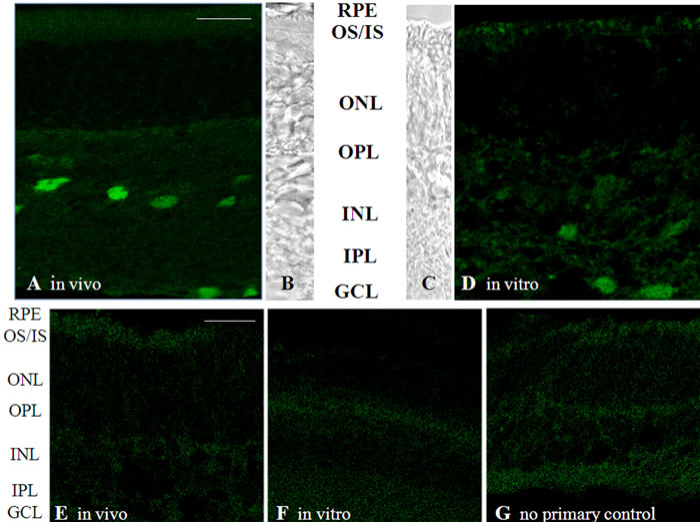
Light-induced signal transmission to the inner retina. After complete dark adaptation, animals and organ cultures were exposed to 1 Hz stroboscopic illumination for 2 h. This procedure examines whether the inner retina can respond to a sustained signal from the photoreceptors. **A**: C-fos expression was induced by stroboscopic illumination in cells in the inner nuclear layer and the retinal ganglion cell layer of the intact animal. **D**: In organ cultures, c-fos expression could also be demonstrated using this paradigm. Images are representative examples from organ cultures derived from three different litters. For each image, a corresponding bright-field image is provided for orientation (**B** and **C**). In the absence of stroboscopic illumination, no c-fos immunoreactivity was observed in vivo (**E**) or in vitro (**F**). **G**: A no-primary antibody control is provided for nonspecific staining of the secondary antibody. Abbreviations: GCL, ganglion cell layer; INL, inner nuclear layer; IPL, inner plexiform layer; IS, inner segments; ONL, outer nuclear layer; OPL, outer plexiform layer; OS, outer segments. The scale bar represents 20 μm.

To test whether signal transmission occurs in the rod pathway under organ culture conditions, c-fos expression in the inner retina in response to stroboscopic illumination was studied. This method has been used successfully in chicken [22], mouse [23], and rat [24]. Although the mechanism of this effect is unknown, we have confirmed using electroretinography (ERG) recordings that in animals with no b-wave (response of the rod bipolar cells in response to light-driven cessation of glutamate release at the rod ribbon synapse), this c-fos response is eliminated [23]. Furthermore, this response could be detected in the mouse retina as early as P12 [23], the approximate time when the rod-driven b-wave can first be recorded in the ERG [38]. Mice (P18) and retina organ cultures (P7+11DIV) were exposed to ~1 Hz stroboscopic illumination for 2 h, and c-fos was identified by immunohistochemistry. As shown previously [23], this illumination induced c-fos expression in ganglion cells and cells in the proximal inner nuclear layer (likely amacrine cells; Figure 4A) of the intact retina. Stroboscopic illumination also induced c-fos expression in the retina grown in organ culture (Figure 4B), although not to the same degree as in the in vivo retina, indicating that rod to inner retina communication is established by this time in the ex vivo retina.

In summary, the RPE in vitro was found to produce between ~40 and 25% of the level of 11-*cis* retinal during the 11 days in culture when compared to the levels generated in vivo. This reduced amount of chromophore was found to be sufficient to support cone development with appropriate targeting of cone outer segment membrane proteins to the outer segments. In addition, sufficient rhodopsin is regenerated in the retina organ cultures to initiate light-mediated signal transmission in the rod pathway, based on the presence of an inner retina response (c-fos expression) to prolonged stroboscopic illumination. The c-fos results, which showed that signal transmission from rods to bipolar cells does occur and is strong enough to be detected, also revealed that culture conditions provide all the essential components to promote expression of the proteins required for cellular development and synaptic transmission within the rod pathway. In future experiments we wish to utilize whole-retina ERG recordings [39], which would allow us to not only quantify the size of the light-induced photoreceptor and ensuing rod bipolar cell responses, but also to compare rod versus cone responses. Finally, the observations that 11-*cis* retinal formation plateaus after 7DIV and both rod and cone OS are stunted suggests that while retinal development occurs in culture, maturation of the retina is arrested in culture. This may ultimately limit the useful range of the culture system to 7DIV.

### Conclusion

Rodent retinas can easily be grown in culture conditions. We established a retina–RPE system of explants from the early postnatal mouse eye to set a defined mimic of the in vivo condition in which mechanisms of photoreceptor development, outer segment membrane protein trafficking, and light-induced signal transmission can be studied. Since many compounds have teratogenic effects, this system will allow us to study compounds in isolation.
